# Triangular Index Ratio as an Alternative Method to the Alpha Angle for Defining the Presence of Cam Morphology: A Prospective Cohort Study

**DOI:** 10.1002/acr.80052

**Published:** 2026-05-18

**Authors:** Jinchi Tang, Fleur Boel, Michiel M. A. van Buuren, Noortje S. Riedstra, Myrthe A. van den Berg, Jos Runhaar, Sita Bierma‐Zeinstra, Rintje Agricola

**Affiliations:** ^1^ Erasmus MC University Medical Center Rotterdam Rotterdam the Netherlands

## Abstract

**Objective:**

Cam morphology, which is a significant risk factor for hip osteoarthritis, is commonly quantified by the alpha angle (AA). This study aims to explore the potential of the triangular index ratio (TIR) to quantify cam morphology on anteroposterior radiographs by assessing the association between TIR‐defined cam morphology and the development of radiographic hip osteoarthritis (RHOA) at 10‐year follow‐up. Additionally, this study compares the predictive performance of cam morphology as defined by the TIR with AA for RHOA development.

**Methods:**

The Cohort Hip and Cohort Knee study included 1,002 participants recruited from the Dutch population aged 45 to 65 years who presented with first‐onset hip or knee pain or both to the general practitioner. The associations between cam morphology hips free of RHOA at baseline (Kellgren and Lawrence [KL] grade <2) and the development of RHOA (KL ≥2) were estimated using logistic regression adjusted for age, sex, and body mass index. Sensitivity, specificity, positive predictive value (PPV), and negative predictive value of the two measurements (TIR ≥1.05 and AA ≥ 60°) for predicting RHOA were evaluated, respectively.

**Results:**

TIR‐defined cam morphology was associated with the development of RHOA at follow‐up with an adjusted odds ratio of 2.48 (95% confidence interval [CI] 1.38–4.46). The PPVs of cam morphology defined by TIR (70.6%, 95% CI 59.0–80.0) and AA (62.3%, 95% CI 53.8–70.1) overlapped, as did their sensitivity (TIR: 7.8%, 95% CI 5.8–10.2; AA: 12.3%, 95% CI 9.8–15.2).

**Conclusion:**

Cam morphology defined by a TIR ≥1.05 at baseline is associated with the development of RHOA 10 years later. TIR may offer a definition of cam morphology more closely related to RHOA, whereas AA may avoid losing too many target cases. Both methods can therefore be used to define cam morphology.

## INTRODUCTION

Cam morphology, a hip shape variation characterized by an osseous prominence at the anterolateral femoral head‐neck junction, has been recognized as a significant morphologic risk factor for hip osteoarthritis.^1^, ^2^, ^3^ Currently, there is no consensus on the gold standard for detecting or quantifying cam morphology. The alpha angle (AA) has emerged as the most widely used measurement because of its relative simplicity and wide applicability to various radiographic views and planes in both 2D and 3D imaging.^1^, ^4^, ^5^ It serves as a measure of the extent of asphericity of the femoral head, with a higher value indicating a greater deviation toward an aspherical femoral head. Previously, a threshold of AA ≥60° was recommended to define the presence of cam morphology.^6^, ^7^

**SIGNIFICANCE & INNOVATIONS**

- The Cohort Hip and Cohort Knee cohort includes 1,002 participants aged 45 to 65 years with early‐stage symptoms in the knee and/or hip and a 10‐year follow‐up. This makes the cohort ideal for studying the development of hip osteoarthritis.
- The triangular index ratio (TIR) is a novel method achieving the same objective as the alpha angle, quantifying the deviation from a spherical to an aspherical femoral head. However, differences in measurement indicators between the two methods result in distinct outcomes. Investigating these differences holds significant potential for guiding the selection of appropriate methods to define cam morphology based on specific research or clinical objectives in the future.
- The TIR could serve as a promising alternative to the alpha angle (AA) for quantifying cam morphology with higher positive predictive values. The AA and TIR each have their advantages in defining the radiographic hip osteoarthritis‐relevant cam morphology.

However, the AA also has limitations. First, considerable variability was observed in its reliability previously. The interobserver intraclass correlation coefficient (ICC) ranged from 0.05 (95% confidence interval [CI] −0.36 to 0.44)^8^ to 0.92 (95% CI 0.89–0.95).^9^, ^10^ Second, the AA only identifies the point where the femoral head‐neck junction begins to protrude and is therefore potentially unable to capture the most prominent portion of the cam morphology. Consequently, a small sliver of protruding bone may result in a similar AA value as a large bump. Additionally, the AA captures a broad range of “aspherical femoral heads,” some of which may not be relevant for the development of hip osteoarthritis.^11^ These findings suggest that using AA alone to define cam morphology may yield inaccuracies.

The triangular index ratio (TIR) is an alternative method for quantifying cam morphology on an anteroposterior (AP) radiograph. Different than the AA, it quantifies cam morphology by measuring the extent to which a potential cam morphology laterally deviates at a predefined point at the head‐neck junction. Given that the AA and TIR measure different aspects, the TIR may serve as an alternative measurement to AA. However, there is a lack of studies using TIR to quantify cam morphology and how this measure relates to the AA.

The aim of this explorative study is to investigate the association between cam morphology defined by TIR and the development of radiographic hip osteoarthritis (RHOA) at follow‐up. Additionally, we aim to assess and compare the predictive performance of the AA and TIR for RHOA development.

## METHODS

### Study population

The study population was drawn from the Cohort Hip and Cohort Knee (CHECK) study, a longitudinal multicenter prospective cohort study in the Netherlands. It included 1,002 individuals aged between 45 and 65 years. These participants were experiencing their first episode of hip and/or knee complaints and had not sought medical assistance or initiated consultations with a general practice within six months before entry. Those who had any other pathologic condition that could explain the symptoms were excluded. The detailed inclusion and exclusion criteria of the CHECK study have been previously outlined.^12^, ^13^ Participants underwent visits at baseline (year 0) and at years 2, 5, 8, and 10 after inclusion. In this study, we focused on the development of RHOA and included only individuals who met the following criteria: availability of both baseline and 10‐year follow‐up radiographs, complete baseline demographic data, and no evidence of RHOA at baseline. Ethical approval for the study was granted by the medical ethics committees of all participating centers, and written informed consent was obtained from all participants.

### Radiographic measurements

Standardized weight‐bearing AP radiographs of the pelvis or hip were obtained at baseline and 10‐year follow‐up. Both AP pelvis and hip radiographs used the same acquisition protocol; participants were positioned with 15° internal rotation of their hips, leading to contact between the medial side of the distal part of the big toes.

The AA is the angle between the femoral neck axis and a line connecting the femoral head center and the alpha point (red point in Figure 1), where the contour of the femoral head‐neck junction begins to leave the best‐fitting circle of the femoral head.^14^ A previously validated threshold value of at least 60° was used to define the presence of cam morphology.^6^

**Figure 1:**
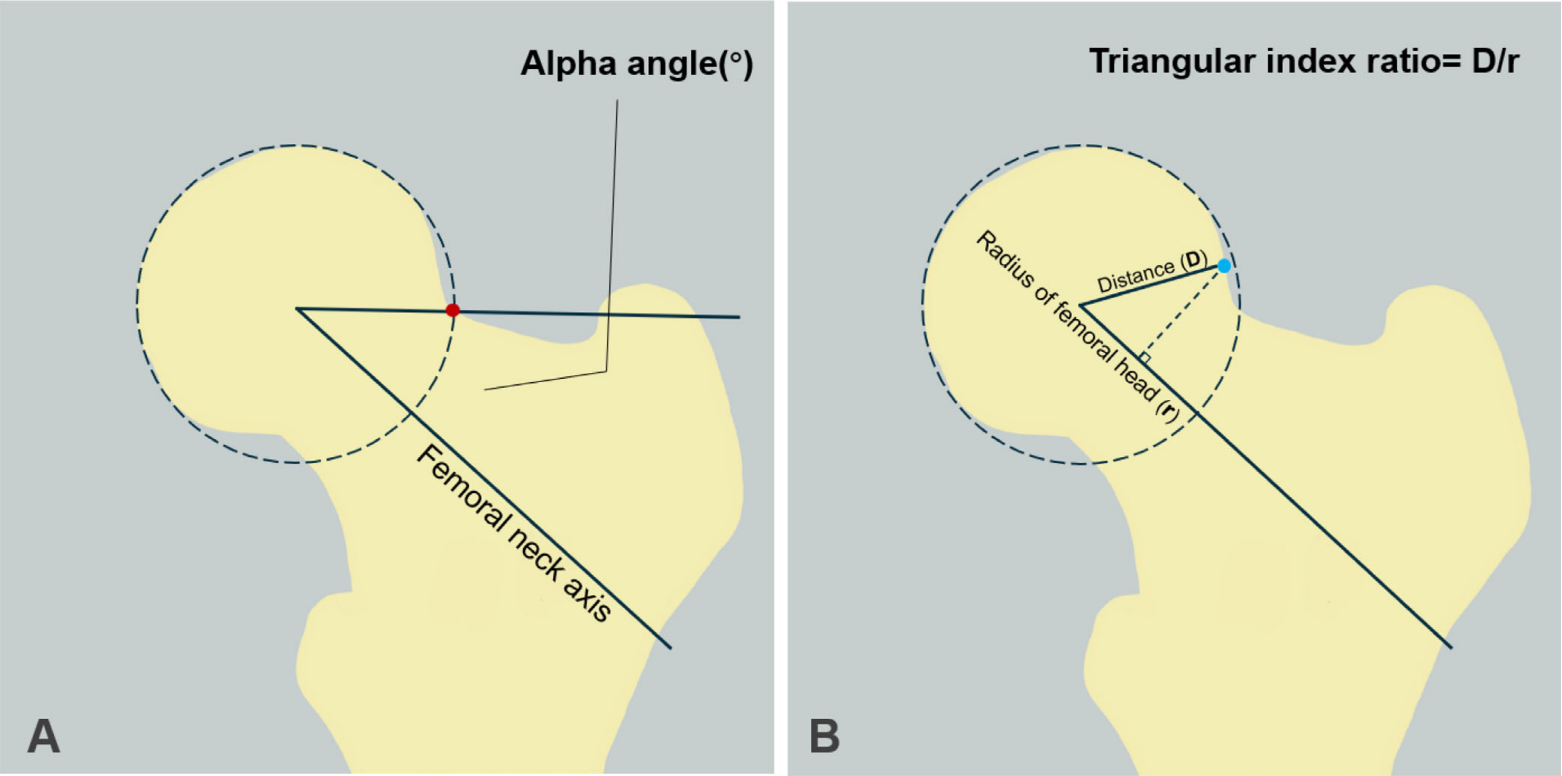
The measurement of AA (A) and TIR (B). AA, alpha angle; TIR, triangular index ratio.

By applying the Pythagorean law for triangular figures, the triangular index defines cam morphology by comparing a specific distance (D) and the radius of femoral head (r).^15^ This distance (D) is measured from the center of the femoral head to the point (blue point in Figure 1) on the superior cortex of the head‐neck junction. The blue point is located on the perpendicular (dotted) line drawn at half the radius of the femoral head along the line passing through the femoral neck axis. Initially, the asphericity of femoral head (cam morphology) was defined by Gosvig et al if D ≥ r + 2 mm at 1.2 magnification.^15^ However, this measurement relies on radiographs achieving millimeter‐level accuracy, with varying magnifications depending on the radiographic protocol used. To address this issue, we converted this linear measurement into a ratio. Based on the original triangular index equation, both sides of the equation were divided by r, giving D/r ≥1 + 2 mm/r. To translate the 2 mm threshold into a dimensionless ratio, we used an average femoral head diameter of 57.16 (± 0.66) mm in the White population (corresponding to r ≈ 28.6 mm).^16^ At 1.2 magnification, 2 mm corresponds to ~0.058 r. Thus, the equation becomes approximately D/r ≥1.058. After rounding to two decimals, we selected the nearest lower cut‐off (1.05) as the threshold value for TIR, consequently defining cam morphology when the ratio D/r ≥1.05.

Both the AA and TIR were automatically and uniformly calculated by an open‐access, in‐house developed, and validated Python script (version 3.7.0) on the baseline radiographs.^17^ This method previously showed good intermethod reliability when compared with manual measurement by two experienced orthopedic surgeons on dual‐energy X‐ray absorptiometry images: 0.81 (95% CI 0.46–0.92) for AA and 0.96 (95% CI 0.91–0.98) for TIR. In comparison, the inter‐ and intra‐observer ICCs were 0.77 (95% CI 0.58–0.89) and 0.89 (95% CI 0.78–0.95) for AA and 0.94 (95% CI 0.89–0.97) and 0.81 (95% CI 0.61–0.91) for TIR.^17^

### Radiographic hip osteoarthritis

The status of RHOA was scored according to Kellgren and Lawrence (KL) classification. For each participant, the radiographs from baseline and all follow‐ups were assessed simultaneously. The development of RHOA was defined by a KL grade ≥2 or a total hip replacement (THR) at 10‐year follow‐up from those having KL grade <2 at baseline.^18^

### Statistical analysis

Univariate differences in baseline characteristics between included and excluded hips were investigated by the independent sample's *t*‐test or chi‐square test. To study the association between cam morphology (defined by TIR ≥1.05 or AA ≥60°) at baseline and the development of RHOA at 10‐year follow‐up, a logistic regression model was used. This regression analysis was performed with generalized estimating equations (GEE) because GEE adjusts for within‐person correlation of left and right hips. The analysis was adjusted for age, sex, and body mass index (BMI), and the strength of association was expressed in odds ratios (OR) with 95% CIs. Sensitivity regression analyses were performed using the same modeling approach but with the outcome redefined to include only incident RHOA cases with KL grade ≥2, excluding hips that underwent THR during follow‐up. To explore the cross‐sectional association between cam morphology and baseline hip pain, the same analytical methods were applied.

An a priori power analysis was performed specifically for the two primary regression models evaluating the associations of TIR ≥1.05 and AA ≥60° with RHOA development. The statistical power was calculated based on the variance of the estimated log‐OR using the Hsieh et al formula.^19^ Power analysis indicated that a minimum total sample size of 826 (TIR model) or 1,280 (AA model) would be sufficient to provide 90% power to detect an association at a two‐sided α = 0.05. We did not perform post hoc power calculations because the achieved sample size (n = 1,416 hips) exceeded the requirements determined by the a priori analysis for both primary models.

To assess the predictive performance for the development of RHOA at follow‐up of cam morphology defined by AA or TIR, we calculated sensitivity, specificity, positive predictive value (PPV), and negative predictive value (NPV) and their 95% CIs. Positive likelihood ratio (LR+) and negative likelihood ratio (LR−) were calculated from the sensitivity and specificity values. Sensitivity analyses were conducted to assess predictive performance using TIR thresholds of at least 1.04, 1.03, and 1.02. All statistical analyses were performed in SPSS Statistics, version 26.0 (IBM Corp., Armonk). The effect was considered significant at *P* < 0.05.

## RESULTS

### Population

Detailed baseline characteristics of the participants and their hips are presented in Table 1. Of the 2,004 hips from 1,002 individuals in the CHECK cohort, 588 hips were excluded for the following reasons: definite RHOA at baseline (n = 240, 12.0%); insufficient baseline radiographic quality preventing automated measurement (n = 111, 5.5%); missing baseline BMI data (n = 2, 0.1%); and lack of 10‐year KL grade data due to loss at follow‐up (n = 235, 11.7%). All missingness was random and not systematically related to other participant characteristics. In total, 1,416 hips from 426 participants were included (mean age: 55.6 ± 5.2 years; 81.7% female; mean BMI: 26.2 ± 4.0). Of these, 330 participants contributed two hip observations and 96 participants contributed one. The complete flow of participants (hips) is provided in the flowchart (Figure 2).

**Table 1:** Baseline characteristics of included and excluded hips from the CHECK cohort*

Baseline characteristics	CHECK cohort		
	Included hips (N = 1,416)	Excluded hips (N = 586)	*P* value
Age, mean (SD), years	55.6 (5.2)	56.6 (5.2)	0.403
Women, n (%)	1,157 (81.7)	423 (71.9)	<0.001
BMI, mean (SD)	26.2 (4.0)	26.1 (4.0)	0.395
AA, mean (SD), degrees	46.4 (9.9)	50.9 (13.7)	<0.001
TIR, mean (SD)	0.96 (0.05)	0.74 (0.43)	<0.001

* AA, alpha angle; BMI, body mass index; CHECK, Cohort Hip and Cohort Knee; TIR, triangular index ratio.

**Figure 2:**
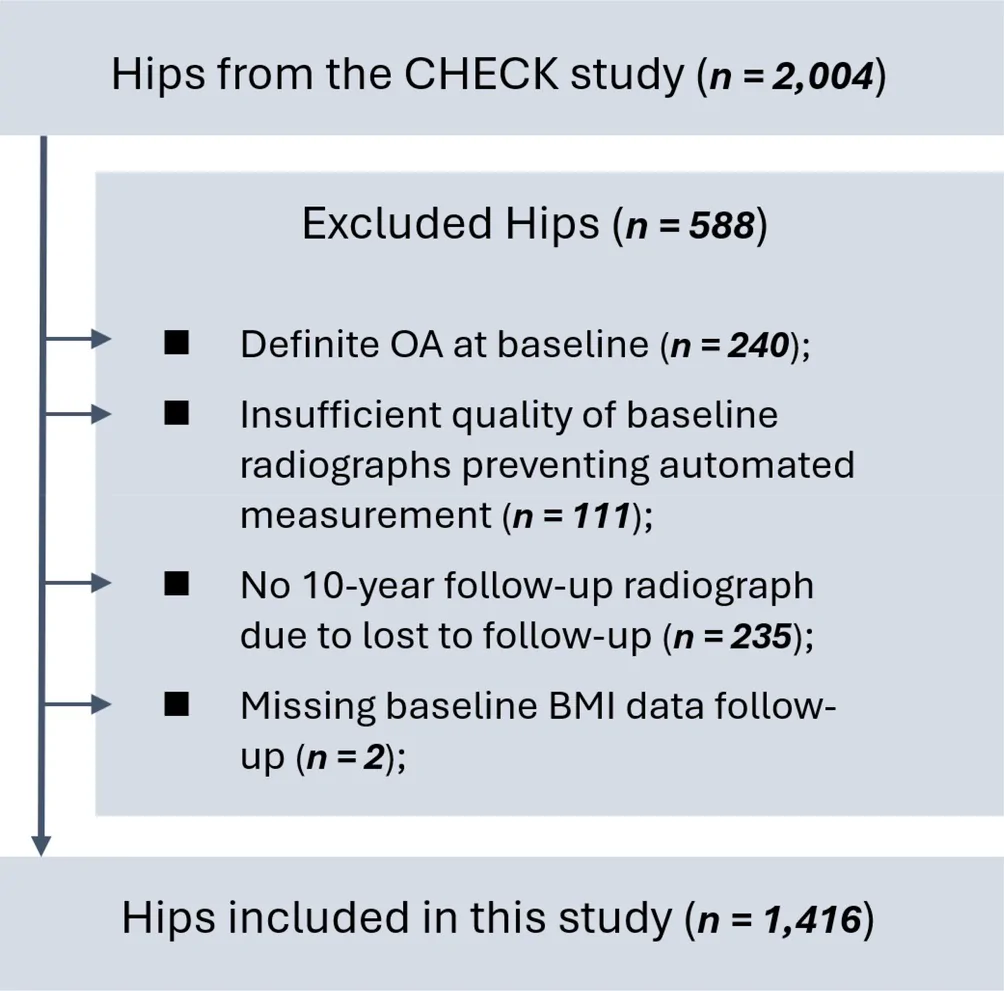
The flow of hips in this study. BMI, body mass index; CHECK, Cohort Hip and Cohort Knee; OA, osteoarthritis. Color figure can be viewed in the online issue, which is available at http://onlinelibrary.wiley.com/doi/10.1002/acr.80052/abstract.

At baseline, there were 122 hips (8.6%) with cam morphology defined by AA ≥ 60° and 68 hips (4.8%) with cam morphology defined by TIR ≥ 1.05. Among them, 65 hips (4.6%) met both cam morphology definitions. The distribution of values for two measurements is shown in Figure 3. Differences in baseline characteristics between hips with cam morphology defined by AA or TIR are shown in Supplementary Table 1.

**Figure 3:**
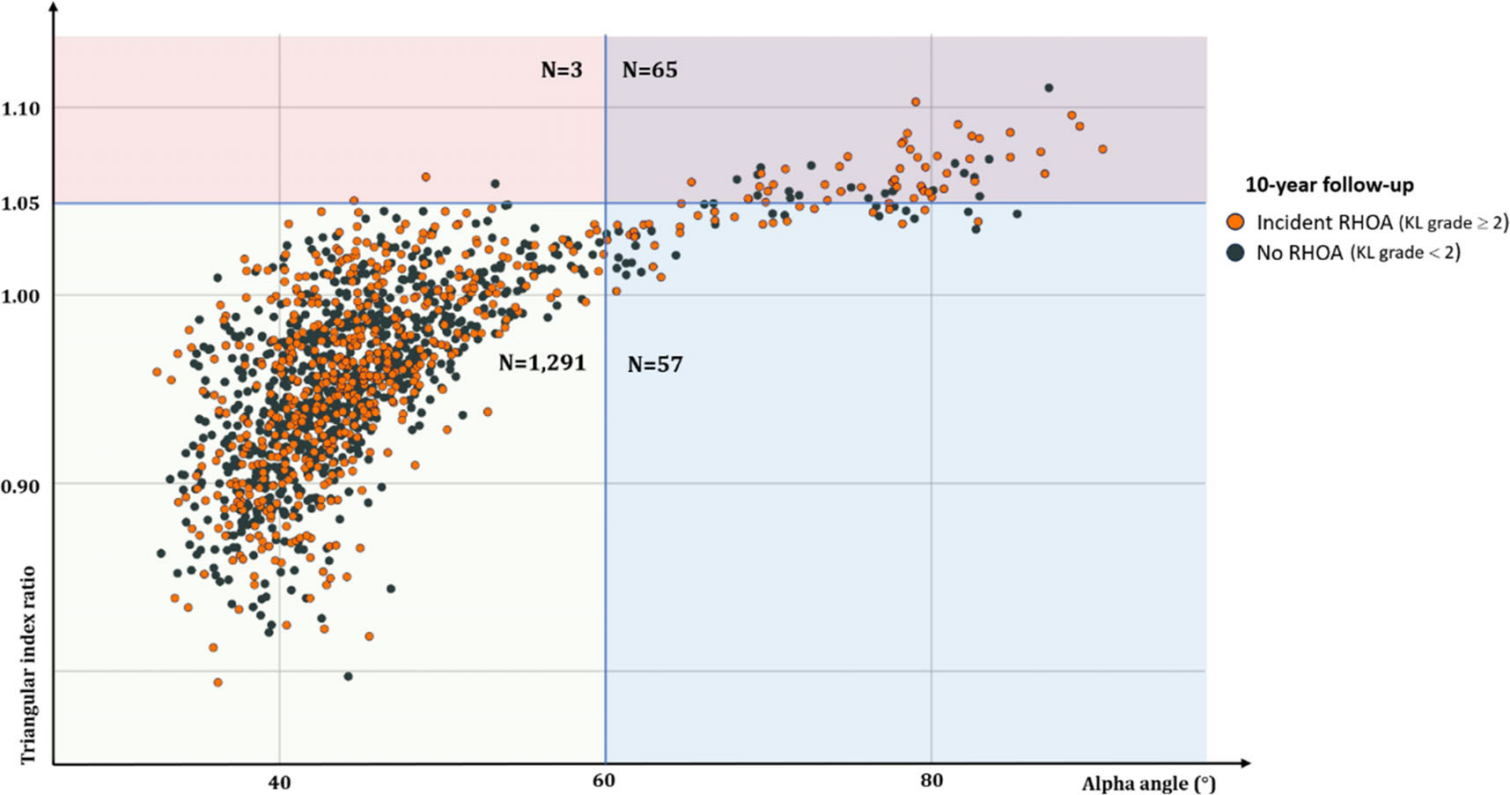
The distribution of included hips according to their value of AA and TIR with outcome at 10‐year follow‐up. Two reference lines, indicating the thresholds of two measurements, divided hips into four categories (blue/red area: hips with cam morphology only fulfilling the AA or TIR threshold; purple area: hips with cam morphology fulfilling both thresholds; green area: hips without cam morphology not fulfilling either threshold). AA, alpha angle; KL, Kellgren and Lawrence; RHOA, radiographic hip osteoarthritis; TIR, triangular index ratio. Color figure can be viewed in the online issue, which is available at http://onlinelibrary.wiley.com/doi/10.1002/acr.80052/abstract.

The incidence of RHOA at 10‐year follow‐up was 43.6% (40.1% when excluding THR cases). Subgroup incidence of RHOA was 70.8% in hips with AA ≥60° and TIR ≥1.05; 52.6% in hips with AA ≥60° and TIR <1.05; 41.8% in hips with AA <60° and TIR <1.05; and 66.6% in hips with AA <60° and TIR ≥1.05 (only three cases in this subgroup).

Cam morphology, defined by TIR ≥1.05 at baseline, was significantly associated with the development of RHOA at 10‐year follow‐up, with an unadjusted OR of 2.90 (95% CI 1.69–4.96) and an adjusted OR of 2.48 (95% CI 1.38–4.46). The unadjusted OR for the method of AA ≥60° is 1.92 (95% CI 1.30–2.83) and the adjusted OR is 1.68 (95% CI 1.10–2.55). No significant cross‐sectional association between cam morphology and baseline hip pain was observed (Supplementary Table 2). The results of the sensitivity analysis are shown in Supplementary Table 3.

The PPV for developing RHOA was 70.6% (95% CI 59.0–80.0) in hips with cam morphology defined by TIR ≥1.05 and 62.3% (95% CI 53.8–70.1) in those defined by AA ≥60°. The NPV was similar within the two methods (AA: 58.2%, 95% CI 57.4–59.0; TIR: 57.8%, 95% CI 57.2–58.4). The sensitivity and specificity of AA were 12.3% (95% CI 9.8–15.2) and 94.3% (95% CI 92.4–95.8), respectively. The corresponding values for TIR ≥1.05 were 7.8% (95% CI 5.8–10.2) and 97.5% (95% CI 96.2–98.5). Both measurements have low LR− (AA: 0.93; TIR: 0.95), whereas TIR (3.12) has slightly higher LR+ than AA (2.16). The results of the sensitivity analysis for the predictive performances of TIR ≥1.04, 1.03, or 1.02 are shown in Table 2.

**Table 2:** Predictive performances of cam morphology defined by AA or TIR for the development of RHOA at follow‐up*

Method for defining CAM morphology	Number of incident RHOA, %	Sensitivity, 95% CI	Specificity, 95% CI	PPV, 95% CI	NPV, 95% CI	Accuracy, 95% CI	LR+, 95% CI	LR−, 95% CI
AA								
≥60°	76 (62.3)	12.3% (9.8–15.2)	94.2% (92.4–95.8)	62.3% (53.8–70.1)	58.2% (57.4–59.2)	58.6% (55.9–61.1)	2.14 (1.51–3.04)	0.93 (0.90–0.96)
<60°	541 (41.8)							
TIR								
≥1.05	48 (70.6)	7.8% (5.8–10.2)	97.5% (96.2–98.5)	70.6% (59.0–80.0)	57.8% (57.3–58.5)	58.4% (55.8–61.0)	3.11 (1.86–5.18)	0.95 (0.92–0.97)
<1.05	569 (42.2)							
≥1.04	64 (63.4)	10.4% (8.1–13.1)	95.4% (93.7–96.7)	63.4% (53.9–71.9)	58.0% (57.2–58.7)	58.3% (55.7–60.9)	2.24 (1.52–3.31)	0.94 (0.91–0.97)
<1.04	553 (42.1)							
≥1.03	92 (63.9)	14.9% (12.2–18.0)	93.5% (91.6–95.1)	63.9% (56.2–71.0)	58.7% (57.8–59.6)	59.3% (56.6–61.8)	2.29 (1.66–3.17)	0.91 (0.88–0.95)
<1.03	525 (41.3)							
≥1.02	120 (59.4)	19.45% (16.4–22.8)	89.7% (87.4–91.7)	59.4% (53.0–65.5)	59.1% (56.0–60.2)	59.1% (56.5–61.7)	1.90 (1.46–2.46)	0.90 (0.86–0.94)
<1.02	497 (40.9)							

* AA, alpha angle; CI, confidence interval; LR+/LR−, positive/negative likelihood ratio; NPV, negative predictive value; PPV, positive predictive value; RHOA, radiographic hip osteoarthritis; TIR, triangular index ratio.

## DISCUSSION

This prospective cohort study showed a significant association between cam morphology defined by TIR ≥1.05 and the development of RHOA within 10 years follow‐up. As an alternative method of AA for defining cam morphology, TIR offers both advantages and disadvantages. This makes each method better suited for different research or clinical purposes.

Although the original definition of TIR was initially proposed as a radiologic index for evaluating femoral head asphericity in 2005,^15^ it has seen limited application in past research. In contrast, the AA, which serves the same purpose, is commonly used. This is likely because of the simplicity of measuring the AA and the ease with which it can be applied to various views of plain radiographs and multiplanar imaging.^20^, ^21^ Conversely, measuring the TIR involves more complex procedures, and there is no evidence that the TIR can be used on radiographic views other than the AP view.

The limitations of the AA are becoming increasingly apparent. With the widespread adoption of automated measurement, the practicality of the AA measurement is becoming less of an advantage. In addition, a recent study reported an intermethod ICC of 0.46 (95% CI 0.12–0.70) for AA compared with 0.78 (95% CI 0.59–0.89) for TIR.^10^ This variance can be attributed to the different measurement procedures for both measurements. The value of AA is defined by the position of the alpha point, which is related to the position of the best‐fitting circle of the femoral head. In contrast, the measurement of TIR is less influenced by this factor.^10^ Moreover, we previously identified several distinct subtypes of cam morphology defined by an AA ≥60°, in which only some were relevant to RHOA development. These findings suggest that relying solely on the AA to define cam morphology may not be suitable for research aiming to efficiently identify high‐risk subgroups for RHOA through the presence of cam morphology.

The TIR is a promising alternative method for quantifying cam morphology. Our result of the logistic GEE model indicates that hips with cam morphology defined by the TIR with a threshold value of ≥1.05 have 2.5 times higher odds (95% CI 1.38–4.46) of developing RHOA at 10‐year follow‐up compared with hips without cam morphology. As a comparison, the ORs for AA in the same population at the same follow‐up is 1.68 (95% CI 1.10–2.55). The TIR therefore can be seen as a valuable method for quantifying cam morphology and identifying those at high risk of developing RHOA. Because the TIR focuses on the distance from the most lateral part of the femoral head‐neck junction to the femoral head center, slight deviations from a spherical femoral head, which may result in higher AA values, will not be classified as cam morphology with the TIR. In our results, we identified 122 cases with AA‐defined cam morphology, whereas only 68 cases were identified by TIR. A total of 46.7% (57 of 122) of cases with AA‐defined cam morphology had TIR values ranging from 1.00 to 1.05, indicating no to slight prominence at the femoral head‐neck junction. Sensitivity analyses using TIR thresholds of 1.04, 1.03, and 1.02 showed that lowering the threshold increased sensitivity and reduced specificity. PPV decreased slightly, and NPV remained similar. These results indicate that the predictive performance of TIR is generally stable, supporting the use of a TIR ≥1.05 as a reasonable threshold that balances overall predictive performance.

From the distribution of two measurements, both of which aim to serve the same purpose, a positive linear correlation between the two values seems to be observed within the included hips. There was some overlap between values of the AA and the TIR, with 65 hips having both an AA ≥60° and a TIR ≥1.05. These overlapping cases have a high deviation to an aspherical femoral head and also have obvious lateral prominence at the femoral head‐neck junction, fitting the description of cam morphology well. There were three cases captured by TIR ≥1.05 but with AA <60°, indicating the contour of the femoral head‐neck junction exceeded but then re‐entered the best‐fitting circle of the femoral head. Although such cases are rare and lack an obvious or typical cam morphology, it is not reasonable to classify them as normal based solely on the AA.

The AA and TIR each have their advantages in predicting the development of RHOA. The PPV for RHOA incidence was somewhat higher when using TIR (70.6%) compared with AA (62.3%), whereas the sensitivity was higher for AA (12.3%) than the obtained TIR (7.8%). Given the overlapping confidence intervals, these findings should be interpreted cautiously, but they suggest that TIR‐defined cam morphology seems more closely related to RHOA, whereas AA may capture a larger proportion of cases. In addition, the slightly higher LR+ indicates that a positive TIR is somewhat more useful than AA for identifying groups with high risk for RHOA. The difference between the predictive performance of the two measurements primarily stems from a substantial portion of hips having an AA ≥60° but a TIR <1.05. The incidence of RHOA in this subgroup (52.6%) is considerably lower than that in hips with AA ≥60° and TIR ≥1.05 (70.8%) but is higher than in normal hips (AA <60° and TIR <1.05) (41.8%). Therefore, TIR ≥1.05 provides a definition of cam morphology more closely related to RHOA, although it overlooks some RHOA‐relevant cases. In contrast, AA ≥60° can capture more target cases, although this leads to lower accuracy. Depending on the goal of the research, the two methods can have different uses. Using the AA could reduce the risk of missing relevant RHOA cases during screening. Conversely, TIR can more precisely identify individuals at high risk of RHOA, aiding in early intervention decisions.

In the sensitivity analysis excluding hips that underwent THR during follow‐up, the incidence of RHOA decreased modestly, and the associations between cam morphology (TIR or AA) and incident RHOA were weakened and no longer statistically significant. This attenuation likely reflects the clinical reality that THR represents a common and unavoidable outcome of progressive RHOA, particularly among individuals with structural abnormalities such as cam morphology. Removing these cases therefore omits a clinically meaningful subset of individuals who would otherwise meet criteria for end‐stage disease, leading to an underestimation of the true association. The findings from the sensitivity analysis support the robustness of our primary findings and reinforce the rationale for including THR in the development of RHOA definition, consistent with previous large cohort studies. This approach helps capture the full spectrum of disease progression—from no radiographic OA change to end‐stage joint failure requiring surgical intervention.

Our study has several limitations. First, the threshold value of the TIR used lacked extensive support from previous studies, given the limited application of the TIR. The threshold we used, TIR ≥1.05, showed good predictive performance compared with AA and other TIR thresholds but still needs external validation. Several population‐based cohorts—such as the Rotterdam Study,^22^ the Osteoarthritis Initiative,^23^ and Multicenter Osteoarthritis Study^24^—include hip imaging data and could potentially facilitate external validation of the TIR‐based cam morphology assessment. Future work using these cohorts, where imaging compatibility allows, would be valuable to confirm and extend our findings. Second, our findings cannot be generalized to a younger or asymptomatic population. Participants in the CHECK cohort study experienced first‐onset symptoms in either their hip or knee or both and were aged between 45 and 65 years at baseline. Consequently, future research should focus on externally validating our findings across different demographic groups. Third, residual confounding by unmeasured factors such as physical activity levels or occupational exposures cannot be fully excluded because direct information on these variables was not collected in the CHECK cohort.

In conclusion, baseline cam morphology defined by a TIR ≥1.05 is associated with the development of RHOA 10 years later and showed a somewhat higher positive predictive values than the method of AA ≥60°. The TIR could potentially serve as a promising alternative to AA for quantifying cam morphology in future research.

## AUTHOR CONTRIBUTIONS

All authors contributed to at least one of the following manuscript preparation roles: conceptualization AND/OR methodology, software, investigation, formal analysis, data curation, visualization, and validation AND drafting or reviewing/editing the final draft. As corresponding author, Dr Agricola confirms that all authors have provided the final approval of the version to be published and takes responsibility for the affirmations regarding article submission (eg, not under consideration by another journal), the integrity of the data presented, and the statements regarding compliance with institutional review board/Declaration of Helsinki requirements.

## Supporting information


**Disclosure form**.

**Supplementary Table 1:** Baseline characteristics of hips with cam morphology defined by alpha angle or triangular index ratio.
**Supplementary Table 2:** Cross‐sectional association between cam morphology and baseline hip pain.
**Supplementary Table 3;** Sensitivity analysis excluding THR cases from the RHOA outcome definition
