# Supplementary material for: Triangular Index Ratio as an Alternative Method to the Alpha Angle for Defining the Presence of Cam Morphology: A Prospective Cohort Study
Source: Arthritis Care Res (Hoboken). 2026 May 18;78(11):1751–8. doi: 10.1002/acr.80052 (PMC13710786; doi:10.1002/acr.80052)
Supplement: Supplementary file 2 [file ACR-78-1751-s002.docx]

**Supplementary Table 1.** Baseline characteristics of hips with cam morphology defined by alpha angle or triangular index ratio.

| Baseline characteristics | Hips with cam morphology | | |
| --- | --- | --- | --- |
|  | Defined by AA≥60° (N=122) | Defined by TIR≥1.05 (N=68) | P value |
| Age in years: mean (SD) | 56.4(5.2) | 57.5(5.1) | 0.159 |
| Women, No (%) | 59(48.4) | 35(51.5) | 0.795 |
| BMI, kg/m²: mean (SD) | 27.4(4.0) | 28.1(4.5) | 0.287 |
| Alpha angle, °: mean (SD) | 73.2(8.2) | 76.5(8.3) | 0.009 |
| Triangular index ratio: mean (SD) | 1.05(0.02) | 1.07(0.01) | <0.001 |
| Hip pain, No (%) | 50(41.0) | 33(48.5) | <0.001 |

*AA: alpha angle; TIR: triangular index ratio; SD: standard deviation; BMI: body mass index.*

**Supplementary Table 2.** Cross-sectional association between cam morphology and baseline hip pain.

| **Cam morphology** | **Unadjusted OR** | **P value** | **Adjusted OR** | **P value** |
| --- | --- | --- | --- | --- |
| TIR ≥ 1.05 | 1.48(0.88-2.50) | 0.14 | 1.67(0.96-2.89) | 0.07 |
| AA ≥ 60° | 1.08(0.73-1.59) | 0.70 | 1.20(0.79-1.82) | 0.39 |

*AA: alpha angle; TIR: triangular index ratio*

**Supplementary Table 3.** Sensitivity analysis excluding THR cases from the RHOA outcome definition

| **Cam morphology** | Outcome: the development of RHOA at follow-up (KL≥2) | | | |
| --- | --- | --- | --- | --- |
|  | Crude OR (95%CI) | P value | Adjusted OR (95%C) | P value |
| TIR≥1.05 | 1.7 (1.0-2.8) | 0.045 | 1.4 (0.8-2.4) | 0.223 |
| AA ≥ 60° | 1.5 (1.0-2.2) | 0.044 | 1.3 (0.8-1.9) | 0.261 |

*OR: Odds ratio; RHOA: radiographic hip osteoarthritis; THR: total hip replacement; TIR: triangular index ratio; AA: alpha angle.*
